# TCP Transcription Factors at the Interface between Environmental Challenges and the Plant’s Growth Responses

**DOI:** 10.3389/fpls.2016.01930

**Published:** 2016-12-21

**Authors:** Selahattin Danisman

**Affiliations:** Molecular Cell Physiology, Faculty of Biology, Bielefeld UniversityBielefeld, Germany

**Keywords:** transcription factor, TCP, development, evolution, plant hormones, signaling

## Abstract

Plants are sessile and as such their reactions to environmental challenges differ from those of mobile organisms. Many adaptions involve growth responses and hence, growth regulation is one of the most crucial biological processes for plant survival and fitness. The plant-specific TEOSINTE BRANCHED 1, CYCLOIDEA, PCF1 (TCP) transcription factor family is involved in plant development from cradle to grave, i.e., from seed germination throughout vegetative development until the formation of flowers and fruits. TCP transcription factors have an evolutionary conserved role as regulators in a variety of plant species, including orchids, tomatoes, peas, poplar, cotton, rice and the model plant *Arabidopsis.* Early TCP research focused on the regulatory functions of TCPs in the development of diverse organs via the cell cycle. Later research uncovered that TCP transcription factors are not static developmental regulators but crucial growth regulators that translate diverse endogenous and environmental signals into growth responses best fitted to ensure plant fitness and health. I will recapitulate the research on TCPs in this review focusing on two topics: the discovery of TCPs and the elucidation of their evolutionarily conserved roles across the plant kingdom, and the variety of signals, both endogenous (circadian clock, plant hormones) and environmental (pathogens, light, nutrients), TCPs respond to in the course of their developmental roles.

## Discovery of TCPs – of *Peloria* and Other Mutants

Developmental plasticity is important for plant survival because plants are sessile organisms that have to adapt to suboptimal environmental conditions. It is crucial that these developmental adaptions are balanced, which means that multiple environmental stimuli have to be perceived and weighed against each other before a plant adjusts its growth. Hence, a plethora of regulatory proteins is involved in governing developmental responses to the environment. One family of transcription factors that is involved in multiple developmental processes are the TEOSINTE BRANCHED 1, CYCLOIDEA, PCF1 (TCP) proteins.

The common toadflax (*Linaria vulgaris*) is a perennial plant with bilateral, zygomorphic flowers that is native to Europe and large parts of northern Asia. When Carl Linnaeus was presented with a common toadflax that did not exhibit zygomorphic but radially symmetric flowers, he called it *peloria* from the Old Greek πέλωρ (pelór), which means *monster*. Linnaeus speculated that this monster was a hybrid between the common toadflax and a thitherto unknown plant and he was surprised to see that this hybrid was nevertheless able to propagate through seeds (Linnaeus and Rudberg, 1744). Whereas his hybrid hypothesis proved to be wrong, he used this case as evidence against immutability, the belief that all species are created at the beginning of the world and are unchanging (Smith, 1821). *Peloria* is a natural variation that occurs in toadflax, snapdragons (*Antirrhinum majus*) (Darwin, 1868) and in foxgloves (*Digitalis purpurea*) (Keeble et al., 1910), amongst other species.

About 250 years later, Luo et al. (1996) isolated the *CYCLOIDEA (CYC)* gene which is only expressed in the dorsal parts of the snapdragon flower and which is responsible for the regulation of zygomorphic flowers. A double mutant of *CYC* and its close homolog *DICHOTOMA* leads to radially symmetric snapdragon flowers (Luo et al., 1996). Cubas et al. (1999b) found that a homolog of the *CYC* gene was also responsible for floral symmetry in the common toadflax. Here, they could show that the *CYC* gene in peloric mutants was extensively methylated and silenced (Cubas et al., 1999b). At about the same time, Doebley et al. (1995) analyzed two quantitative trait loci that control morphological differences between domesticated maize (*Zea mays*) and its wild progenitor teosinte. They found the *teosinte branched 1* (*tb1*) mutation, which leads to increased side shoot outgrowth, and showed that the difference between the maize and the teosinte variant of *TB1* lies mainly in the regulatory regions of the gene, i.e., whereas the function remains the same, the expression pattern is different between domesticated maize and teosinte (Wang et al., 1999).

Kosugi et al. (1995) found that two promoter motifs that are important for the transcriptional regulation of the proliferating cell nuclear antigen (PCNA) gene in rice (*Oryza sativa*) were bound by two transcription factors that were designated PCF1 and PCF2 (Kosugi and Ohashi, 1997). Finally, Cubas et al. (1999a) determined that the above described proteins **T**B1, **C**YC and **P**CF1 and PCF2 share a conserved non-canonical bHLH region, the eponymous TCP domain (Kosugi and Ohashi, 1997).

## Form and Function of TCP Transcription Factors

Whereas, this review will mainly focus on the evolutionarily conserved roles of TCPs in the regulation of plant development and their interactions with endogenous and environmental signals, it is crucial to understand how they function. TCP transcription factors are divided into two classes, class I and class II TCPs. These classes differ in the composition of their respective NLSs, the length of the second helix in the bHLH domain, and the presence of an arginine-rich domain of unknown functionality outside the bHLH domain (Cubas et al., 1999a). This so-called R domain is not found in class I TCPs and was predicted to form a hydrophilic α-helix or a coiled-coil structure that mediates protein–protein interactions (Lupas et al., 1991; Cubas et al., 1999a).

The basic region of the TCP domain is essential for DNA binding. Replacement of a conserved glycine–proline pair in the basic region by two lysines completely abolished DNA binding activity of TCP4 in electrophoretic mobility shift studies (Aggarwal et al., 2010). Addition of the major groove binding dye methyl green reduced TCP4 binding to DNA, indicating that TCP4 binds to the major groove in double stranded DNA (Aggarwal et al., 2010).

In various experimental approaches, class I and class II TCP proteins have been shown to recognize GC-rich sequences in target gene promoters (Kosugi and Ohashi, 1997; Li et al., 2005; Viola et al., 2011; Danisman et al., 2012). The differences between class I and class II binding preferences are dependent on the presence of glycine or aspartic acid at positions 11 or 15, respectively (Viola et al., 2012). Interestingly, the class I and class II consensus binding site sequences are not mutually exclusive, indicating that at least a subset of potential target genes are targeted by both class I and class II TCP proteins. This led to speculations about a possible antagonistic relation between class I and class II TCPs, where these proteins compete for common target genes and inhibit or activate gene expression depending on which class dominates the target gene promoter (Li et al., 2005). So far, this was shown in one case only, where the *Arabidopsis* class I TCP transcription factor TCP20 binds to the same promoter as the class II TCP4 and regulates the target gene *LIPOXYGENASE2(LOX2)* in the opposite direction to TCP4 (Danisman et al., 2012). It is likely though that more cases of class I-class II TCP antagonisms will be discovered in the future, as the two classes are frequently discovered to be involved in the same biological processes.

Similar to many transcription factor families, TCPs require dimerization to bind to DNA, as addition of deoxycholate, an inhibitor of protein–protein interactions, to electrophoretic mobility shift assays leads to a reduction of TCP binding to target sequences (Trémousaygue et al., 2003). Dimerization between TCP transcription factors first has been described between PCF1 and PCF2 in rice, which form homo- and heterodimers (Kosugi and Ohashi, 1997). Whereas the homodimer of TCP20 for example does not bind to the promoter of the iron homeostasis regulator *BHLH39* in yeast one-hybrid experiments, the TCP20 heterodimer with TCP8 or TCP21 does (Andriankaja et al., 2014). A systematic yeast two-hybrid approach between *Arabidopsis* TCPs found that many protein–protein interactions are possible between TCPs and that there is a preference to bind to TCPs of the own class, i.e., class I TCPs preferably interact with class I TCPs and class II TCPs preferably interact with class II TCPs (Danisman et al., 2013). Dimerization of TCPs are facilitated by IDR (Valsecchi et al., 2013). These are characterized by low compactness, low globularity and higher structural flexibility and are typically present extensively in eukaryotic transcription factors (Liu et al., 2006). The C-terminal IDR of TCP8 is needed for self-assembly of TCP8 in dimers and higher order complexes. These IDRs potentially facilitate the flexibility of TCPs in the choice of interacting partners and thus increases the number of potential functions TCP transcription factors can be involved in Thieulin-Pardo et al. (2015). TCPs not only interact with TCPs: protein–protein interactions with a plethora of other proteins has been described, including negative regulators of effector-triggered immunity (Kim et al., 2014), components of the circadian clock (Pruneda-Paz et al., 2009, 2014; Giraud et al., 2010), and others (Trémousaygue et al., 2003; Tao et al., 2013).

## Evolutionary Conserved Roles of TCPs

The three eponymous TCP proteins were characterized as regulators of branching, floral symmetry, and the cell cycle (Doebley et al., 1995; Luo et al., 1996). Later, both CYC-like and the PCF-like TCPs were shown to be involved in leaf development (Kosugi and Ohashi, 1997; Palatnik et al., 2003). TCP research since then has focused on these three developmental processes, mainly identifying evolutionarily conserved processes in a wide array of plant species and the role of cell cycle regulation in the observed phenotypes. Recently it became clear however that TCPs are not limited to branching, floral symmetry and leaf development, and neither are they limited to cell cycle mediated regulation of growth. Both will be discussed further below.

TEOSINTE BRANCHED 1, CYCLOIDEA, PCF1 transcription factors belong to an evolutionary conserved family that first appears in fresh water algae of the *Charophyta* family (Navaud et al., 2007). In the bryophyte *Physcomitrella patens*, knockout of the TCP transcription factor *Pp*TCP5 leads to increased numbers of sporangia that are attached to a single seta, reminiscent of **branching** phenotypes of *tcp* mutants in higher land plants (Ortiz-Ramírez et al., 2016). Hence, control of meristematic activity of axillary meristems with a subsequent effect on branching patterns seems to be an ancient role of TCP transcription factors (Ortiz-Ramírez et al., 2016). Consistent with this finding, branching phenotypes are apparent both in monocot and dicot plant species. Overexpression of the rice OsTB1, an ortholog of maize TB1, led to a strong decrease in tiller number. The number of axillary buds was not affected in these plants but their outgrowth was Takeda et al. (2003). This fits to the observation that it is not the formation of axillary meristems but the outgrowth of these that is affected by TCPs (Braun et al., 2012). This has been shown in peas (Braun et al., 2012), poplar (Muhr et al., 2016), *Arabidopsis* (Aguilar-Martínez et al., 2007; Poza-Carrión et al., 2007) and potato (Nicolas et al., 2015).

TCP effect on **floral development** was shown in a wide range of plant species, including *Arabidopsis*, Antirrhinum, annual candytuft (*Iberis amara*) (Busch and Zachgo, 2007; Busch et al., 2012), angiosperms like *Aristolochia arborea* and *Saruma henryi* (Horn et al., 2015), Gerbera species (Broholm et al., 2008), rice (Yuan et al., 2009), sunflowers (Fambrini et al., 2012), peas (Wang et al., 2008), ragworts (Kim et al., 2008), Morrow’s honeysuckle (*Lonicera morrowii*) (Howarth and Donoghue, 2006), *Knautia macedonica* (Berger et al., 2016), and orchids (De Paolo et al., 2015).

Phylogenetic analysis revealed that the CYCLOIDEA-like TCPs underwent two major duplication events that both predate the formation of core eudicots (Howarth and Donoghue, 2006). In *Arabidopsis*, all three CYC clades are represented by TCP12, TCP1 and TCP18, respectively (Howarth and Donoghue, 2006). Especially the CYC2 clade, represented by TCP1 in *Arabidopsis*, underwent multiple additional duplications and has been studied for its effect on floral symmetry, as it contains the original *CYC* gene of Antirrhinum (Howarth and Donoghue, 2006). An interesting side note is that the duplication of the CYCLOIDEA-like TCPs nearly coincides with the major duplication events of the homeotic MADS-box transcription factors APETALA3, AGAMOUS and SEPALLATA, all three of them important factors for the definition of organ identity in flowering plants (Howarth and Donoghue, 2006). This suggests that the genetic components that are important for the definition of floral organs diversified at a similar time as the components that are important for the growth regulation of these. TCP transcription factors have been identified as targets of *Arabidopsis* APETALA1 and SEPALLATA3 (Kaufmann et al., 2009, 2010), highlighting a possible link between organ identity formation and growth regulation between MADS-box transcription factors and TCPs (Dornelas et al., 2011).

In Antirrhinum, CYC regulates symmetry via the Myb-domain transcription factor RADIALIS (Corley et al., 2005). Overexpression of CYC in *Arabidopsis* leads to larger petals containing enlarged petal cells (Costa et al., 2005). Regulation of floral growth is not restricted to the CYC-like class II TCPs. In the *jaw*-D mutant, petal development is different from wild type *Arabidopsis* (Palatnik et al., 2003). Nag et al. (2009) showed that this depends on miR319 regulation of *TCP4*. A microRNA-resistant form of *TCP4* under the control of an *APETALA3* promoter is expressed in floral organs only and leads to dramatically smaller flowers that only consist of carpels and sepals, missing any petals or stamens, whereas the seedlings of these plants look normal (Nag et al., 2009).

The zinc-finger transcriptional repressor RABBIT EARS controls the expression of the TCPs *TCP5, TCP13*, and *TCP17* and misexpression of both *RABBIT EARS* and these *TCPs* leads to aberrant petal development in *Arabidopsis* (Huang and Irish, 2015). Repression of these TCPs leads to an early stop of mitotic activity during petal development (Huang and Irish, 2015). Interestingly the opposite occurs upon downregulation of *TCP5, TCP13*, and *TCP17* in leaves, where leaf cells continue with mitotic divisions for a longer time than in wild type plants (Efroni et al., 2008). Here, the effect of TCP transcription factors on organ development is dependent on the organ-context. This underlines the importance of the regulatory interplay between TCPs and organ identity regulators. While there are hints at this interplay between TCPs and MADS box transcription factors during flower development, such an interplay remains to be shown during the development of other organs (Dornelas et al., 2011).

First indications for a role of TCPs in **leaf development** comes from work in Antirrhinum (Nath et al., 2003). The Antirrhinum class II TCP mutant *cin* displays crinkly leaves, which are the result of a change in the regulation of the cell cycle during leaf development (Nath et al., 2003). Essentially, mitotic divisions of developing leaf cells in the leaf tip are arrested first and those at the leaf base are arrested last. The result of this successive arresting behavior is a so called arrest front that moves from the leaf tip to the leaf base. The form of this arrest front is different in *cin* leaves than in wild type leaves, leading to a modified leaf curvature (Nath et al., 2003). In *Arabidopsis*, similar behavior is observed in the *jaw*-D mutant (Palatnik et al., 2003). *Jaw*-D is an overexpressor of the microRNA miR319a in which the *CIN*-like class II TCPs *TCP2, TCP3, TCP4, TCP10*, and *TCP24* are downregulated (Palatnik et al., 2003). *Jaw*-D mutants display serrated leaves, abnormal petals and delayed leaf development and senescence (Palatnik et al., 2003). This phenotype derives from delayed leaf development, in which the mitotic arrest front starts later than in wild type plants (Efroni et al., 2008). Recently, it was shown that miR319a-regulated TCP transcription factors act redundantly with NGATHA transcription factors to limit meristematic activity of leaf meristems during leaf development (Alvarez et al., 2016). This phenotype was also apparent in plants expressing an artificial microRNA against the class II TCPs *TCP5, TCP13*, and *TCP17* and the phenotype was extremely strong when these plants were crossed with *jaw*-D plants (Efroni et al., 2008).

Class II TCPs also regulate leaf development in tomato compound leaves. An ortholog of the *Arabidopsis* miR319-sensitive TCPs in tomato is *LA* and it is under the control of the tomato miR319 (Ori et al., 2007). *La* mutants exhibit simple leaves, whereas overexpression of miR319 without *LA* insensitivity to the microRNA leads to increased partitioning of the compound leaves. Also, miR319 overexpressing tomato leaves grow 3 months longer than wild type leaves and show the marks of late differentiation, which is a behavior that is identical to *Arabidopsis jaw*-D plants (Ori et al., 2007; Efroni et al., 2008). Overexpression of miR319 in the monocot *Agrostis stolonifera* (creeping bentgrass) leads to downregulation of class II TCPs and to the formation of wider and thicker leaves that are different from the wild type (Zhou et al., 2013). This phenotype stems from an increased number of cells in the transgenic bentgrass, similar to *jaw*-D in *Arabidopsis* (Efroni et al., 2008; Zhou et al., 2013). In general, expression of CIN-like genes is closely correlated with leaf shapes both in Solanaceae species and in the desert poplar (*Populus euphratica*) (Shleizer-Burko et al., 2011; Ma et al., 2016).

Expression of TCP3 with a dominant repressor domain led to severe disturbance of *Arabidopsis* development in all organs (Koyama et al., 2007), involving ectopic shoot formation, serrated leaves, modified sepals and petals, and wavy silique formation. This was due to misexpression of boundary specific genes, i.e., *CUC* and *LATERAL ORGAN BOUNDARIES* (Koyama et al., 2007). Also in Antirrhinum, an ortholog of *Arabidopsis* TCP15 was found to interact with CUPULIFORMIS, a protein that is related to *Arabidopsis* CUC proteins (Weir et al., 2004). Furthermore, the two *Arabidopsis* class I TCPs TCP14 and TCP15 were shown to be redundant in affecting cell proliferation during leaf development and in other tissues in *Arabidopsis*. The most obvious effect though was seen in internode length, which is reduced in *tcp14 tcp15* mutants and leads to shorter plants (Kieffer et al., 2011).

**FIGURE 1:**
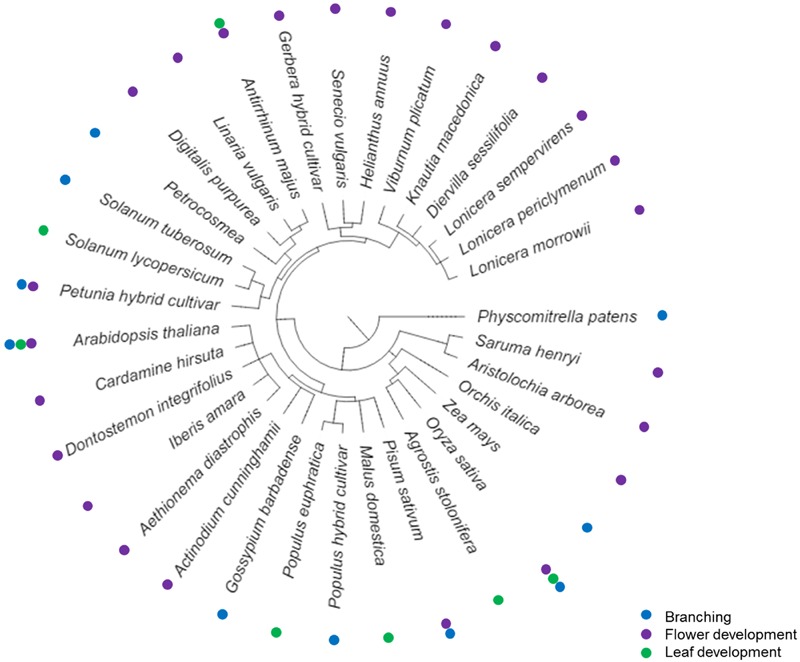
**Phylogenetic tree of plant species in which TCP transcription factors are involved in branching (Takeda et al., 2003; Aguilar-Martínez et al., 2007; Poza-Carrión et al., 2007; Bai et al., 2012; Braun et al., 2012; Drummond et al., 2015; Nicolas et al., 2015; Muhr et al., 2016) (blue dots), flower development (Linnaeus and Rudberg, 1744; Keeble et al., 1910; Corley et al., 2005; Costa et al., 2005; Busch and Zachgo, 2007; Broholm et al., 2008; Kim et al., 2008; Nag et al., 2009; Yuan et al., 2009; Howarth et al., 2011; Busch et al., 2012; Tähtiharju et al., 2012; Claßen-Bockhoff et al., 2013; Juntheikki-Palovaara et al., 2014; De Paolo et al., 2015; Horn et al., 2015; Lucero et al., 2015; Wang et al., 2008; Wang X.et al., 2015; Yang et al., 2015; Berger et al., 2016) (purple dots) or leaf development (Kosugi and Ohashi, 1997; Nath et al., 2003; Palatnik et al., 2003; Koyama et al., 2007, 2010a,b; Ori et al., 2007; Efroni et al., 2008; Kieffer et al., 2011; Mimida et al., 2011; Sarvepalli and Nath, 2011; Danisman et al., 2012, 2013; Aguilar-Martínez and Sinha, 2013; Burko et al., 2013; Tao et al., 2013; Zhou et al., 2013; Ballester et al., 2015; Huang and Irish, 2015; Ma et al., 2016) (green dots), respectively.** The phylogenetic tree was created using Phylotree and iTOL (Letunic and Bork, 2016).

Whereas TCP functions have thus been very well-characterized in these branching, flower and leaf development over a wide array of plant species (**Figure 1**), there are hints that this is just a subset of TCP roles in development. TCPs were shown to be upregulated upon imbibition of dry seeds and germination of *tcp14* transposon insertion lines seemed to be lower than in wild type seeds (Tatematsu et al., 2008). Although here, expression of *TCP14* in the transposon lines was not necessarily lower than in the wild type, indicating that *TCP14* may not be the only cause of the reduced germination rate (Tatematsu et al., 2008). Downregulation of *TCP* expression in cotton led to reduced cotton hair fiber length as well as a higher of lateral shoots and a stunted growth indicative of a reduced apical dominance (Hao et al., 2012). Overexpression of miR319 in Chinese cabbage not only led to altered leaf development, also the cabbage heads were rounder than in cabbage with low *miR319* expression and higher expression of its target gene *BrpTCP4-1* (Mao et al., 2014). Heterologous expression of the rice OsTCP19 in *Arabidopsis* led to a lower number of lateral roots (Mukhopadhyay et al., 2015). In cucumber, mutation of a *TCP* gene led to a unique plant phenotype. The affected cucumber plants did not develop tendrils but shoots instead. The authors of this study hypothesize that here TCPs not only affect growth of an organ but also determine organ identity (Wang S.et al., 2015). A similar phenotype was found in melons where a single-nucleotide mutation in *CmTCP1* led to the Chiba tendril-less mutation. Also here, the tendrils were converted to shoot and leaf-like structures (Mizuno et al., 2015). This would be the first indication that TCPs can act as organ identity regulators. Further research has yet to uncover whether the function of TCPs in organ identity regulation of tendrils is a unique and novel role or whether other plant organs also need TCPs to define their identity.

## TCP Functions Effect on the Cell Cycle – Direct or Indirect?

Early, TCP research focused on the cell cycle as main target of TCP regulation (Kosugi and Ohashi, 1997; Li et al., 2005). Whereas, binding to cell cycle genes has been shown in certain cases (Li et al., 2005; Davière et al., 2014), close analysis of cell division patterns and transcript changes during *jaw*-D leaf development indicated that the class II-TCP dependent regulation of the cell cycle may be indirect (Efroni et al., 2008). Also binding of the class I TCP TCP20 to cell cycle genes has been shown only once and *in vitro* (Li et al., 2005), whereas direct target gene analysis indicate that hormone synthesis, especially jasmonate synthesis, is rather directly targeted by TCP20 (Danisman et al., 2012). Both TCP4 and TCP20 affect leaf development via the synthesis of methyl jasmonate, a hormone that has multiple functions in plant development and response to wounding and pathogens (Schommer et al., 2008). Jasmonate, usually known for its role in wounding and pathogen response, does also affect the cell cycle (Świątek et al., 2002).

**FIGURE 2:**
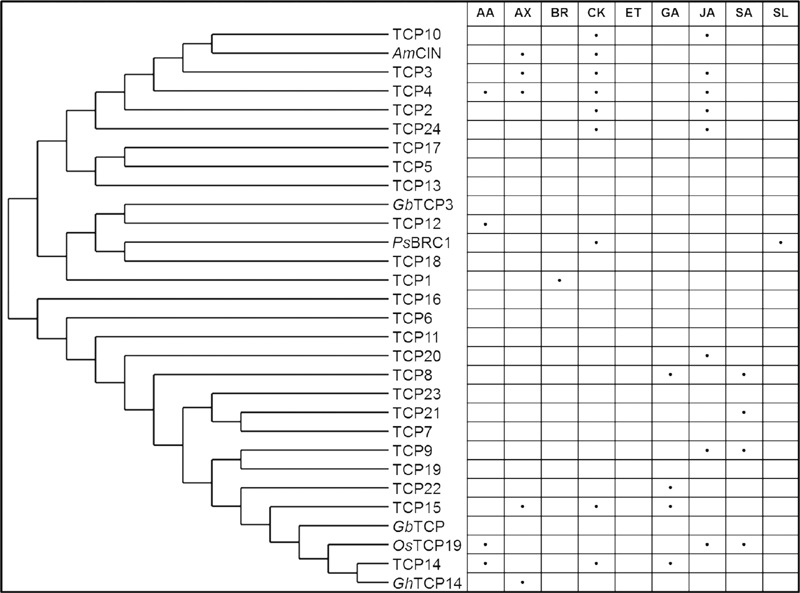
**Hormonal pathways associated with *Arabidopsis* TCP transcription factors and orthologs.** The proteins were plotted according to their phylogenetic similarity using PhyML and TreeDyne (Dereeper et al., 2008). AA, abscisic acid; AX, auxin; BR, brassinosteroids; CK, cytokinin; ET, ethylene; GA, gibberellic acid; JA, jasmonic acid; SA, salicylic acid; SL, strigolactones.

Jasmonate is not the only plant hormone that may mediate TCP regulation to the cell cycle. The evidence for hormone involvement in TCP-mediated growth regulation accumulated in the recent years (Nicolas and Cubas, 2016). TCP functions have been associated with abscisic acid (Tatematsu et al., 2008; González-Grandío et al., 2013; Mukhopadhyay et al., 2015), auxin (Kosugi et al., 1995; Ben-Gera and Ori, 2012; Uberti-Manassero et al., 2012; Das Gupta et al., 2014), brassinosteroid (Guo et al., 2010), cytokinin (Steiner et al., 2012; Efroni et al., 2013), GA (Yanai et al., 2011; Das Gupta et al., 2014; Davière et al., 2014), jasmonic acid (Schommer et al., 2008; Danisman et al., 2012), salicylic acid (Wang X.et al., 2015), and strigolactone signaling pathways (Dun et al., 2012; Hu et al., 2014) (**Figure 2**).

**FIGURE 3:**
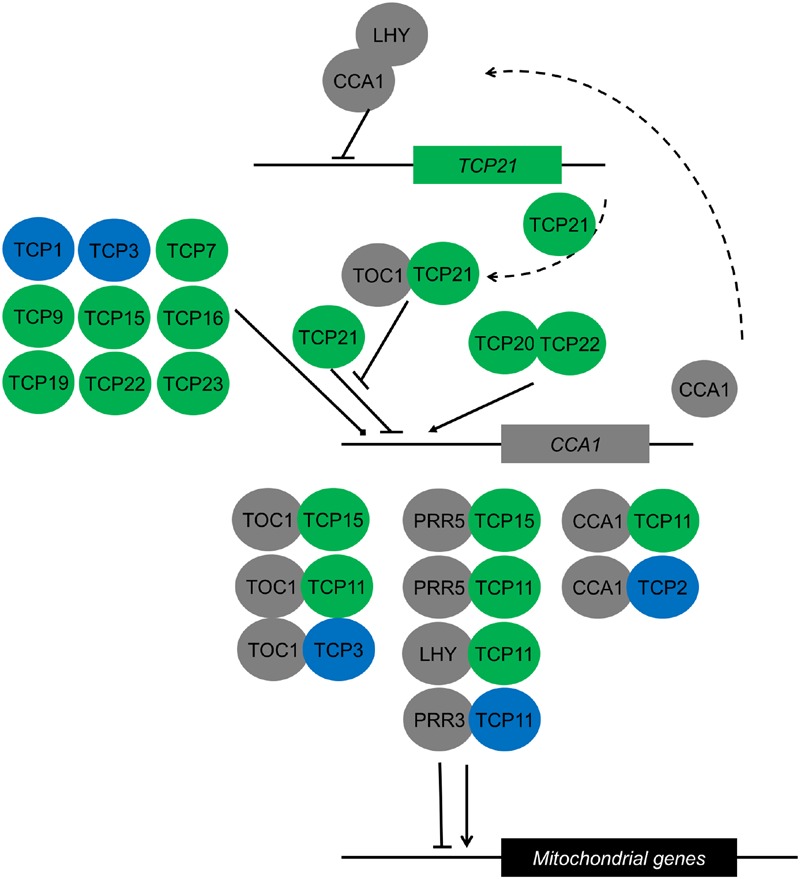
**Interactions of TCP transcription factors with components of the circadian clock both within the central clock circuitry and in downstream processes.** Class I and class II TCPs are depicted in green and blue, respectively. Known clock components are depicted in gray. Proteins are represented as circles, genes in squares. Dimers are depicted as overlapping circles. *CCA1* inhibition by TCP21 is abolished by dimerization of TCP21 with TOC1. The CCA1/LHY dimer inhibits *TCP21* expression (Pruneda-Paz et al., 2009). The effect of nine TCPs that bind to the *CCA1* promoter in yeast one-hybrid studies is unknown (Pruneda-Paz et al., 2014). Downstream of the clock, TCP/clock component heterodimers regulate rhythmic expression of mitochondrial proteins depending on the number and arrangement of TCP binding sites in the mitochondrial gene promoters (Giraud et al., 2010).

Apart from hormonal control of growth, TCP transcription factors are also involved in other biological processes that in turn affect growth. For example, binding sites of TCP transcription factors have been identified in the promoters of *CYTOCHROME C1* and 103 genes that are encoding components of the mitochondrial oxidative phosphorylation machinery and protein biogenesis (Welchen and Gonzalez, 2006). The authors of this study proposed that the TCP transcription factors binding these sites coordinate mitochondria genesis and function with growth in new organs (Welchen and Gonzalez, 2006). Another study showed these genes contain a GGGC(C/T) element in their promoters which is important for diurnal regulation of their gene expression (Giraud et al., 2010). These promoters are bound by TCP transcription factors, implying a role in diurnal regulation of transcripts of the mitochondrial oxidative phosphorylation machinery (Giraud et al., 2010). Earlier TCP21 was found to bind to the promoter of the core clock gene *CCA1* and regulate its expression (Pruneda-Paz et al., 2009). TCP21 serves as an inhibitor of *CCA1* during the day and dimerization of TOC1 with TCP21 abolishes its binding to the *CCA1* promoter. In a double mutant with the clock gene LHY, *tcp21/lhy* greatly reduces the period of *CCA1* expression (Pruneda-Paz et al., 2009). Not only TCP21, other TCPs have also been found to bind to *CCA1* in yeast based studies and co-immunoprecipitation experiments (Giraud et al., 2010; Pruneda-Paz et al., 2014). A recent study also showed that TCP20 and TCP22 act as activators of *CCA1* in the morning, fulfilling an important role in the circuity of the circadian clock (Wu et al., 2016). This means that TCP proteins bind to the promoters of clock genes, regulate their expression, dimerize with clock proteins and bind to downstream targets of the clock (Pruneda-Paz et al., 2009, 2014; Giraud et al., 2010; Wu et al., 2016) (**Figure 3**). Altogether, it becomes clear that TCPs not only affect growth via the cell cycle. Instead, they act in different biological processes that directly or indirectly affect growth.

## Mediating Environmental Signals into Growth Responses

This picture becomes even more complex, as TCPs also mediate environmental signals into growth responses. TCPs were found to be involved in pathogen defense. First, an extensive study showed that both *Pseudomonas syringae* and *Hyaloperonospora arabidopsidis* infection led to reduction of TCP14 protein (Mukhtar et al., 2011). Secreted proteins from pathogenic bacteria transferred by the Aster leafhopper (*Macrosteles quadrilineatus*) to *Arabidopsis* were able to dimerize with and destabilize TCP2, TCP4, and TCP7 proteins, comprising both classes of TCP transcription factors (Sugio et al., 2011, 2014). Overexpression of the responsible phytoplasma protein SECRETED ASTER YELLOWS-WITCHES BROOM PROTEIN 11 in *Arabidopsis* destabilizes TCP2, TCP3, TCP4, TCP5, TCP10, TCP13, TCP17, and TCP24 and leads to *jaw*-D-like phenotypes (Sugio et al., 2011). Additionally, jasmonic acid levels in infected *Arabidopsis* leaves are significantly reduced in comparison with untreated leaves, indicating that the plant’s defense mechanisms are reduced upon infection by the pathogen. A similar effect has been found in apples, where the plant pathogen *Candidatus Phytoplasma mali* binds to two TCP transcription factors and induces morphogenetic changes that co-occur with reduction of jasmonic acid, salicylic acid, and abscisic acid levels (Janik et al., 2016). Further studies identified the class I TCPs TCP8 and TCP9 as important factors for the expression of *ICS1*, which encodes for a key enzyme in salicylic acid synthesis (Wang X.et al., 2015). In another study, TCP21 has been identified to bind to the promoter of *ICS1* and induction of *ICS1* expression by salicylic acid is blocked in *tcp21* mutants (Zheng et al., 2015). Class I TCPs also interact with proteins known to regulate *ICS1* expression, i.e., the transcription factors WRKY28, NAC019 and ETHYLENE INSENSITIVE 1 and the calmodulin binding protein SYSTEMIC ACQUIRED RESISTANCE DEFICIENT 1. Consequently, the *tcp8 tcp9* double mutant shows increased sensitivity to infection with *Pseudomonas syringae* pv. *maculicola* ES4326 (Wang X.et al., 2015). TCP transcription factors partially control pathogen defense via a second pathway, i.e., by antagonizing the effect of SUPPRESSOR OF rps4-RLD1, a protein that negatively regulates effector-triggered immunity in *Arabidopsis* (Kim et al., 2014). Lack of TCPs in the triple mutant *tcp8 tcp14 tcp15* leads to increased growth of *Pseudomonas syringae* DC3000 when compared to wild type plants (Kim et al., 2014).

Recent studies showed that TCP transcription factors regulate flowering time. A knockout of the class I TCP transcription factor TCP23 led to earlier flowering than the wild type, whereas TCP23 overexpressing lines showed delayed flowering behavior (Balsemão-Pires et al., 2013). The floral transition of axillary meristems in *Arabidopsis* is controlled by an interaction between the flowering time proteins FT and TWIN SISTER OF FT and BRC1 (Niwa et al., 2013). The protein–protein interactions between these transcription factors have been shown in yeast two-hybrid, bimolecular fluorescence complementation, and *in vitro* pull-down assays (Niwa et al., 2013). As *brc1* mutants exhibit accelerated flowering and *ft* and *twin sister of ft* mutants exhibit slower flowering of axillary meristems, respectively, it seems that there is an antagonistic relationship between BRC1 and the flowering time proteins (Niwa et al., 2013). It is likely that dimerization of BRC1 with FT and TWIN SISTER OF FT represses their function in axillary meristems (Niwa et al., 2013). The apple FT orthologs *Md*FT1 and 2 were also found to interact with TCP transcription factors (Mimida et al., 2011). Overexpression of the tomato miR319 led to flowering with fewer leaves than in wild type tomato and it was shown that LA binds to the promoters of the tomato *APETALA1* and *FRUITFUL* orthologs (Burko et al., 2013).

Perception of the red to far-red light ratio (R:FR) informs a plant of shading by neighboring vegetation and a lower R:FR ratio leads to suppressed axillary meristem outgrowth, allowing the plant to invest in a longer hypocotyl and eventually avoid the shading. In *Arabidopsis*, hypocotyl elongation is regulated via the bHLH transcription factor PHYTOCHROME INTERACTING FACTOR 4, which among others activates *YUCCA8* expression to promote cell elongation (Sun et al., 2012). *YUCCA2, 5*, and *8* are also direct target genes of TCP4. In fact, induced overexpression of TCP4 leads to elongated hypocotyls and this effect is dependent on both auxin and brassinosteroid signaling (Challa et al., 2016). In potato, *BRC1a* regulation is dependent on the R:FR. BRC1a comes in two forms: the short form (BRC1a^S^) and the alternatively spliced long version (BRC1a^L^). Both result in proteins but the shorter form is cytoplasmatic and does not bind to target genes in the nucleus. The ratio between these two forms changes upon decapitation of potato shoots, exposure to darkness, and under low R:FR conditions (Nicolas et al., 2015). Whereas decapitation leads to a relative increase in BRC1a^S^, darkness and low R:FR treatments lead to a relative increase in BRC1a^L^ content. The longer BRC1a^L^ protein subsequently inhibits axillary branch elongation in potato shoots and stolons (Nicolas et al., 2015). *Arabidopsis brc1* and *brc2* (*tcp12* and *tcp18*) show a reduced response to R:FR and the response is abolished in the *brc1 brc2* double mutant (González-Grandío et al., 2013). TCP transcription factors are also involved in axillary bud outgrowth of Petunia. Here, *Gh*TCP3 acts in conjunction with DECREASED APICAL DOMINANCE 2, a receptor protein that normally inactivates strigolactones in response to decreased R:FR (Drummond et al., 2015). Rice OsTCP15 is involved in the mesocotyl elongation in response to darkness and responds to strigolactone and cytokinin treatments, outlining the interplay between TCPs and different plant hormones in developmental regulation that is responsive to the environment (Hu et al., 2014).

Viola et al. (2013) showed that class I TCPs contain a conserved cysteine-20 which is sensitive to treatments by oxidants in a dose-dependent manner. This redox-dependent behavior of TCP15 is important for its effect in anthocyanin biosynthesis. A mutant in which the cysteine-20 of TCP15 was replaced by a serine accumulates less anthocyanin under high light stress than wild type plants (Viola et al., 2016). Plant extracts from TCP15 overexpressing plants showed that exposure to prolonged high light conditions leads to an abolishment of TCP15 DNA-binding activity *in vivo*, mirroring the *in vitro* phenotype (Viola et al., 2013, 2016). Thus, TCP15 function is reactive to high light input. While the anthocyanin response is not a direct developmental response, further analysis may show that there is a developmental effect.

**FIGURE 4:**
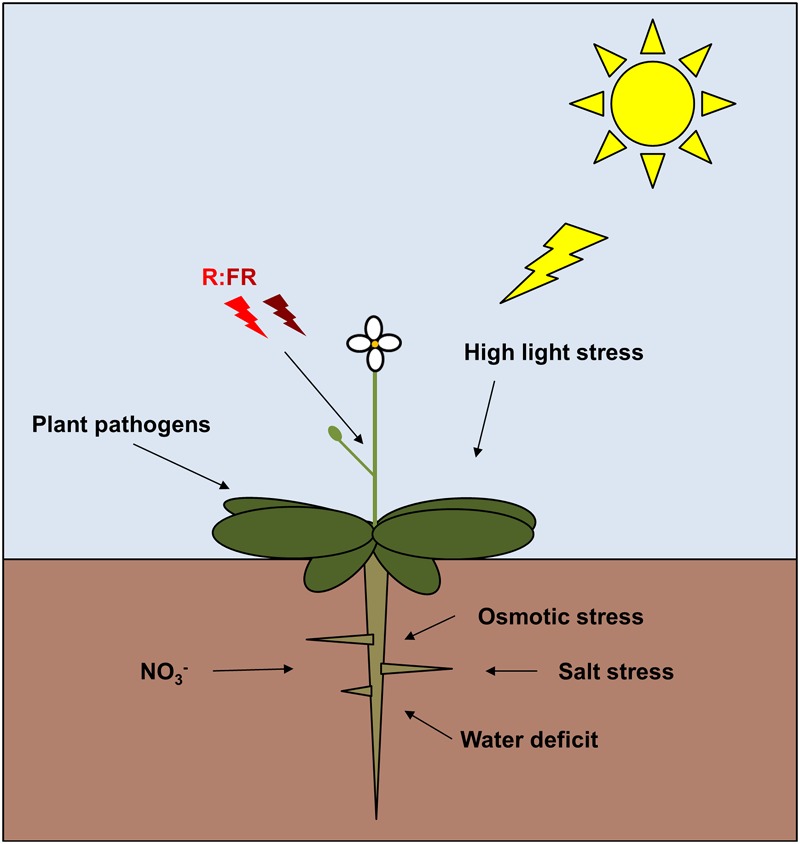
**Schematic figure depicting the diversity of environmental signals that affect TCP functions in plants (Mukhtar et al., 2011; Sugio et al., 2011; Balsemão-Pires et al., 2013; González-Grandío et al., 2013; Niwa et al., 2013; Viola et al., 2013, 2016; Guan et al., 2014; Hu et al., 2014; Kim et al., 2014; Mukhopadhyay et al., 2015; Nicolas et al., 2015; Kumar et al., 2016)**.

Not only light affects TCPs, also other signals are perceived and lead to TCP-mediated growth regulation. For example, Guan et al. (2014) showed that TCP20 is involved in nutrient foraging of *Arabidopsis* roots. In split-root experiments wild type *Arabidopsis* develops an increased number of lateral roots in medium containing high nitrate concentrations (i.e., 5 mM NO_3_^-^) and close to no lateral roots in medium containing low nitrate concentrations (i.e., 0 mM NO_3_^-^). *Tcp20* plants do not exhibit this behavior, indicating that the regulation of root foraging is under the control of TCP20 (Guan et al., 2014). Interestingly, *TCP20* transcript levels are not under the control of nitrate levels, indicating that TCP20 is regulated on protein level, either by forming specific protein–protein dimers in the case of nitrate deficiency or via another regulatory mechanism. In rice, the transcript of the class I TCP *OsTCP19* is upregulated during salt stress and water-deficit treatments (Mukhopadhyay et al., 2015). Heterologous *OsTCP19* overexpression in *Arabidopsis* leads to reduced numbers of lateral roots but increased abiotic stress tolerance, i.e., plants grew better on Mannitol-containing medium and recovered better after water-deficit treatments. Here, *LOX2* expression was reduced in the OsTCP19 overexpressors and ABA signaling genes were upregulated (Mukhopadhyay et al., 2015). Recent experiments revealed up- and down-regulation of several TCPs in *Arabidopsis* under osmotic stress, although a functional analysis of their role in response to osmotic stress has not been done yet (Kumar et al., 2016). In summary, these few results are first indications that TCPs are no mere static regulators of development, but that they do directly translate environmental signals into growth regulation (**Figure 4**).

## Conclusion and Outlook

TCP transcription factors play a role in a multitude of growth processes over a wide range of plant species (**Figure 1**). They affect growth directly via the cell cycle and indirectly via influencing plant hormonal signaling and the circadian clock (**Figures 2** and **3**). Additionally, recent discoveries link TCP-controlled growth responses with environmental signals such as R:FR, high light stress, salt stress or the presence or absence of nutrients.

TCP transcription factors are involved in so many important developmental processes and interact with so many plant hormones that it is likely that future plant research will also uncover a lot more signals that TCPs react to. This will also mean that future TCP research will have to more closely elucidate how the interaction of TCPs with different signaling networks is regulated to ensure a measured response to environmental challenges. This research will have to uncover the roles of dimerization, transcriptional and post-transcriptional regulation as well as post-translational modifications in controlling and ensuring specific TCP functions in plant development.

Plant pathogens are targeting TCP transcription factors to manipulate plant architecture in their favor. If plant pathogens use TCPs in their best interests, maybe so should we. TCP transcription factors will be valuable tools in optimizing plant architecture and hardening plants in response to environmental challenges.

## Author Contributions

SD drafted, wrote and critically revised the article.

## Conflict of Interest Statement

The author declares that the research was conducted in the absence of any commercial or financial relationships that could be construed as a potential conflict of interest.

## Abbreviations

bHLH: basic helix-loop-helix
BRC: BRANCHED
CCA1: CIRCADIAN CLOCK ASSOCIATED 1
CIN: CINCINNATA
CUC: CUP-SHAPED COTYLEDON
CYC: CYCLOIDEA
FT: FLOWERING LOCUS T
GA: gibberellic acid
ICS1: ISOCHORISMATE SYNTHASE 1
IDR: intrinsically disordered region
*jaw*-D: *JAGGED AND WAVY*-D
LA: LANCEOLATE
LHY: LATE ELONGATED HYPOCOTYL
LOX: LIPOXYGENASE
NLS: nuclear localization signal
PRR: PSEUDO RESPONSE REGULATOR
TB1: TEOSINTHE BRANCHED 1
TCP: TEOSINTE BRANCHED 1, CYCLOIDEA, PCF1
TOC1: TIMING OF CAB EXPRESSION1
